# Pathogenic Leptospires Modulate Protein Expression and Post-translational Modifications in Response to Mammalian Host Signals

**DOI:** 10.3389/fcimb.2017.00362

**Published:** 2017-08-09

**Authors:** Jarlath E. Nally, Andre A. Grassmann, Sébastien Planchon, Kjell Sergeant, Jenny Renaut, Janakiram Seshu, Alan J. McBride, Melissa J. Caimano

**Affiliations:** ^1^Infectious Bacterial Diseases Research, National Animal Disease Center, United States Department of Agriculture, Agricultural Research Service Ames, IA, United States; ^2^Biotechnology Unit, Technological Development Center, Federal University of Pelotas Pelotas, Brazil; ^3^Departments of Medicine, Pediatrics, and Molecular Biology and Biophysics, University of Connecticut Health Center Farmington, CT, United States; ^4^Environmental Research and Innovation Department, Luxembourg Institute of Science and Technology Belvaux, Luxembourg; ^5^Department of Biology, University of Texas San Antonia San Antonia, TX, United States; ^6^Gonçalo Moniz Institute, Oswaldo Cruz Foundation, Ministry of Health Salvador, Brazil

**Keywords:** *Leptospira*, spirochetes, proteomics, DIGE, post-translational modifications

## Abstract

Pathogenic species of *Leptospira* cause leptospirosis, a bacterial zoonotic disease with a global distribution affecting over one million people annually. Reservoir hosts of leptospirosis, including rodents, dogs, and cattle, exhibit little to no signs of disease but shed large numbers of organisms in their urine. Transmission occurs when mucosal surfaces or abraded skin come into contact with infected urine or urine-contaminated water or soil. Whilst little is known about how *Leptospira* adapt to and persist within a reservoir host, *in vitro* studies suggest that leptospires alter their transcriptomic and proteomic profiles in response to environmental signals encountered during mammalian infection. We applied the dialysis membrane chamber (DMC) peritoneal implant model to compare the whole cell proteome of *in vivo* derived leptospires with that of leptospires cultivated *in vitro* at 30°C and 37°C by 2-dimensional difference in-gel electrophoresis (2-D DIGE). Of 1,735 protein spots aligned across 9 2-D DIGE gels, 202 protein spots were differentially expressed (*p* < 0.05, fold change >1.25 or < −1.25) across all three conditions. Differentially expressed proteins were excised for identification by mass spectrometry. Data are available via ProteomeXchange with identifier PXD006995. The greatest differences were detected when DMC-cultivated leptospires were compared with IV30- or IV37-cultivated leptospires, including the increased expression of multiple isoforms of Loa22, a known virulence factor. Unexpectedly, 20 protein isoforms of LipL32 and 7 isoforms of LipL41 were uniformly identified by DIGE as differentially expressed, suggesting that unique post-translational modifications (PTMs) are operative in response to mammalian host conditions. To test this hypothesis, a rat model of persistent renal colonization was used to isolate leptospires directly from the urine of experimentally infected rats. Comparison of urinary derived leptospires to IV30 leptospires by 2-D immunoblotting confirmed that modification of proteins with trimethyllysine and acetyllysine occurs to a different degree in response to mammalian host signals encountered during persistent renal colonization. These results provide novel insights into differential protein and PTMs present in response to mammalian host signals which can be used to further define the unique equilibrium that exists between pathogenic leptospires and their reservoir host of infection.

## Introduction

Pathogenic species of *Leptospira* cause leptospirosis, a bacterial zoonotic disease with a global distribution affecting over one million people annually (Costa et al., 2015). Leptospires colonize renal tubules and are excreted in urine from reservoir hosts of infection. Contact with contaminated urine or water sources can result in infection via breaches of the skin and/or mucosal surfaces and disseminate haematogenously, causing a range of clinical symptoms from mild fever, to icteric Weil's disease and pulmonary hemorrhage syndrome. Mortality rates range from 10 to 70% depending on disease severity (McBride et al., 2005). In developed countries, leptospirosis is primarily a recreational disease, an occupational disease of farm workers, veterinarians, and slaughter plant workers, and in returning travelers. In developing countries, it is a socioeconomic disease perpetuated by rapid urbanization, rodent infestation, and transmission via contaminated water sources associated with limited infrastructures and severe weather events. Both rodents and domestic farm animal species serve as reservoir hosts of infection and sources of disease transmission to humans.

A large body of work has demonstrated that leptospires regulate and modify gene expression in response to environmental cues, as encountered during disease transmission, including changes in temperature (Lo et al., 2006; Qin et al., 2006; Matsunaga et al., 2013), osmolarity (Matsunaga et al., 2007), concentration of iron (Lo et al., 2010), the presence of serum (Patarakul et al., 2010), and interaction with macrophages (Xue et al., 2010). However, since leptospires are not readily amenable to genetic manipulation, the functional and biological significance of these transcriptomic changes are unclear (Adler et al., 2011; Picardeau, 2015). There often is limited correlation between gene and protein expression by leptospires (Lo et al., 2009) but it is clear that the protein profiles of pathogenic leptospires also are regulated in response to environmental cues encountered during host infection (e.g., in response to temperature and iron depletion; Nally et al., 2001a,b; Cullen et al., 2002; Eshghi et al., 2009). More recently, it was shown that protein expression, in response to changing environmental conditions, can be modified further by specific post-translational modifications (PTM; Eshghi et al., 2012). Indeed, both saprophytic and pathogenic leptospires have comprehensive bio-systems to modify proteins (Cao et al., 2010; Schmidt et al., 2011; Stewart et al., 2016). Recently, 32 phosphorylated, 46 acetylated, and 155 methylated proteins were identified that not only confirmed multiple modifications in prokaryotes, but also suggests that *L. interrogans* shares significant similarities with protein modification systems in eukaryotes (Cao et al., 2010). The surface-exposed outer membrane protein, OmpL32, undergoes differential methylation of glutamic acid residues in response to modifying *in vitro* growth conditions that emulate those encountered during mammalian host infection (Eshghi et al., 2012). In addition, *L. interrogans* can utilize endogenous biosynthetic pathways to elaborate surface structures containing sialic acids and related nonulosonic acids (Ricaldi et al., 2012b). Finally, the detection of PTM on lysine residues within LipL32 from *in vivo*-isolated *L. interrogans* implies that the infection-generated modification of leptospiral proteins may have a biologically relevant function during the course of infection (Witchell et al., 2014).

The paucibacillary nature of spirochetal infections, combined with the challenges associated with acquiring pathogens free from contaminating host proteins, makes the study of these bacteria in a mammalian host-adapted state inherently difficult. As an alternative approach, we developed a novel animal model in which leptospires are cultivated in a dialysis membrane chambers (DMCs) implanted within the peritoneal cavity of rats, where they are exposed to some of the environmental cues encountered during host infection (Caimano et al., 2014). This strategy has been applied successfully to compare the transcriptome of *L. interrogans* cultivated within DMCs with that of leptospires grown under standard *in vitro* conditions. In addition to determining the relative expression levels of “core” housekeeping genes under both growth conditions, we identified 166 genes that were differentially-expressed at the mRNA level by *L. interrogans* in response to mammalian host signals (Caimano et al., 2014).

In the current study, we applied the DMC model to compare the proteome of *in vivo*-derived leptospires with that of leptospires cultivated *in vitro* at 30°C or 37°C by 2-dimensional difference in-gel electrophoresis (2-D DIGE). Our analysis indicates that the abundance of leptospiral proteins is modulated in response to mammalian host signals, and not temperature alone. In addition, we confirm that in several proteins there is a change in the presence of the PTM trimethyllysine and acetyllysine in response to environmental cues encountered during persistent renal colonization in a reservoir host of infection. These results provide novel insights into the proteome, including PTM, in response to mammalian host signals, which can be used to further define the unique equilibrium that exists between pathogenic leptospires and their reservoir host of infection.

## Materials and methods

### *In vitro*-cultivated bacteria

Virulent low-passage *Leptospira interrogans* serovar Copenhageni strain Fiocruz L1-130, kindly provided by Dr. David Haake (UCLA), was cultivated *in vitro* under standard static conditions for 8–9 days at 30 or 37°C in EMJH medium supplemented with 1% rabbit serum (Pel-Freez Biologicals, Rogers, AR) with 100 μg/ml 5-fluorouracil. Cultures were grown to late logarithmic phase (1–3 × 10^8^ per ml). Virulent low-passage *L. interrogans* serogroup Icterohaemorrhagiae strain RJ19115 was cultivated under standard static conditions at 30°C in EMJH medium until late logarithmic phase (1–3 × 10^8^ per ml).

### Dialysis membrane chamber (DMC)-cultivated bacteria

All animal experimentation was conducted in accordance with protocols as reviewed and approved by the University of Connecticut Health Center Institutional Animal Care and Use Committee. To obtain mammalian host-adapted organisms, virulent low-passage *L. interrogans* serovar Copenhageni strain Fiocruz L1-130 was cultivated in DMCs as previously described (Caimano et al., 2014; Grassmann et al., 2015). In brief, DMCs were prepared with 8–10 ml of EMJH medium [supplemented with 10% vaccine-grade bovine serum albumin (EMD Millipore, Billerica, MA) to maintain osmotic pressure] at a starting inoculum of 10^4^ organisms per ml. Using strict aseptic technique, each DMC was implanted into the peritoneal cavity of an anesthetized female Sprague-Dawley rat (Harlan). Approximately 9–10 days later, DMCs were explanted and the quality of leptospires evaluated for motility and density (1–3 × 10^8^ leptospires per ml) by dark field microscopy using a Petroff-Hausser counting chamber (Hausser Scientific Co., Horsham, PA).

### Urinary derived leptospires

All animal experimentation was conducted in accordance with protocols as reviewed and approved by the Animal Care & Use Committee at the National Animal Disease Center, and as approved by USDA Institutional guidelines. Urinary derived leptospires were collected from experimentally infected rats as previously described with slight modification (Monahan et al., 2008; Bonilla-Santiago and Nally, 2011). Male Sprague-Dawley rats (Harlan) of ~5 weeks of age were experimentally infected with 1 × 10^7^ *L. interrogans* strain RJ19115 by intraperitoneal injection, a stain of *L. interrogans* serogroup Icterohaemorrhagiae which results in significant numbers of leptospires excreted in urine from colonized renal tubules. At 2 weeks post-infection (Rojas et al., 2010), rats were housed overnight in metabolism cages and urine collected directly into a 50 ml conical tube containing 0.5 ml Urine preservative (Norgen Biotek Corp). Urine samples were centrifuged at 4°C, 1,000 × g, for 5 min to remove excess debris. Supernatants were then centrifuged at 6,100 × g, 4°C for 60 min to collect urinary derived leptospires. Pellets were resuspended in 1 ml ice-cold 10 mM Tris-Cl, 1 mM EDTA, transferred to a microfuge tube and collected by centrifugation at 12,000 × g, 4°C for 30 min. Samples were frozen at −20°C until analysis.

### 2-Dimensional fluorescence difference in-gel electrophoresis (2-D DIGE)

2-D DIGE was performed on 18 biological replicates of *L. interrogans* serovar Copenhageni strain Fiocruz L1-130 comprising 6 biological replicates of leptospires cultivated at 30°C, 6 biological replicates of leptospires cultivated at 37°C and 6 biological replicates of leptospires cultivated in DMCs. *In vitro*- and DMC-cultivated leptospires were harvested at late logarithmic phase by centrifugation, washed twice with TE buffer (10 mM Tris-Cl, 1 mM EDTA, pH 7.4) and resuspended in solubilization buffer (7 M Urea, 2 M Thiourea and 1% ASB-14) as previously described (Caimano et al., 2014). Protein concentrations were determined using the DC protein assay kit (Bio-Rad) as per manufacturer's instructions. An internal standard was prepared using a mixture of equal amounts of all 18 replicate samples included in the analysis. This internal standard was included in all gels to allow normalization of each independent gel and cross-gel comparison of each spot's density. 2-D DIGE was performed as previously described (Schuller et al., 2015) using 24 cm IPG strips pH 3–7 NL. Labeled samples were mixed according to the experimental design (Table 1) and the volume adjusted to 150 μl with rehydration buffer comprising 7 M Urea, 2 M Thiourea, 0.5% (w/v) CHAPS and 2% (v/v) ampholytes. After an overnight rehydration of the strips in 450 μl of Destreak Rehydration Solution (GE Healthcare) complemented with 2% ampholytes, the samples were cup-loaded and focused to reach a total of 75 kVh (150 V for 3 h, 300 V for 3 h, a gradient to 1,000 V over 6 h, a gradient to 10,000 V over 3 h, and 10,000 V for 6.25 h). The second dimension was carried out on an HPE system with large 12% non-fluorescent gels (Serva) following manufacturer's instructions.

**Table 1:** 2-D DIGE experimental design.

**Gel #**	**Cy3**	**Cy5**	**Cy2**
1	IV37-Sample 2	DMC-Sample 1	Internal standard
2	IV30-Sample 9	IV37-Sample 7	Internal standard
3	DMC-Sample 2	IV30-Sample 3	Internal standard
4	IV30-Sample 1	DMC-Sample 5	Internal standard
5	IV37-Sample 1	IV30-Sample 8	Internal standard
6	DMC-Sample 3	IV37-Sample 9	Internal standard
7	IV37-Sample 8	DMC-Sample 7	Internal standard
8	IV30-Sample 2	IV37-Sample 3	Internal standard
9	DMC-Sample 6	IV30-Sample 7	Internal standard

*Leptospires were cultured under in vitro conditions at 30°C (IV30) or 37°C (IV37) and compared with leptospires cultured in dialysis membrane chambers (DMC) as described*.

### Image acquisition and analysis

Gels were scanned using the Typhoon® FLA 9500 (GE Healthcare, Buckinghamshire, UK) as per manufacturer's instructions. Gels were analyzed with the Decyder version 7.0. Spot volumes were compared between samples derived from leptospires cultivated at 30°C, 37°C or in DMCs. For each spot, the ratio between conditions is calculated; when the ratio is above one, the fold change equals the ratio and if the ratio is below 1, the fold change is given as −(1/ratio). Statistically significantly expressed protein spots were defined as having a *t*-test *p* < 0.05 and a fold change of >1.25 or < −1.25 (Supplementary Table 1). Spots not meeting these statistical criteria were considered non-differentially expressed.

### Protein digestion and identification

Spots of interest were picked using an Ettan spot picker (GE Healthcare) and digested with a fully automated Evo 2 workstation (Tecan). In brief, excised spots are washed and the proteins reduced with DTT and alkylated with iodoacetamide. After removal of excess salts, proteins were digested for 6 h at 37°C with 40 ng of trypsin in 50 mM ammonium carbonate (TrypsinGold, Promega). After digestion, peptides were extracted from the gel pieces, the extracts dried and spotted onto a MALDI plate. Mass spectra were acquired using a MALDI-TOF-TOF mass spectrometer (Sciex 5800) and after the acquisition of a MS spectrum, the 10 highest peaks (excluding known contaminants) are selected for fragmentation. The MS-spectrum and the 10 MS/MS spectra are grouped in the database search with an in-house MASCOT engine (version 2.3, Matrix Science, matrixscience.com, London, UK). The primary database searched was the “*L. interrogans* serovar Copenhageni str. Fiocruz L1-130” database, downloaded on the 6th of February 2014 from NCBI (txid267671) and containing 7,818 sequences. When high-quality spectra were not matched during this primary search, they were individually resubmitted to a search against the entire NCBInr database (downloaded on the 23rd of September 2013 and containing 32,770,904 sequences). This resulted in the identification of bovine serum albumin (BSA) in some of the spots (Supplementary Table 2), a common contaminant due to the high concentration of BSA in the EMJH used for DMCs. The following parameters were used for the database searches: carbamidomethyl (C) as fixed modification; dioxidation (W), oxidation (M, W), and Trp to kynurenin (W) as variable modifications; peptide mass tolerance of 100 ppm, fragment mass tolerance of 0.5 Da; Trypsin as enzyme with a maximum of 2 missed cleavages. Compliant with minimal requirements for protein identification, no identifications purely based on the MS spectra were accepted. In addition to the requirement that the protein score had to be above the *p* < 0.05 threshold, only protein identifications supported by at least two peptides with a score above the identity threshold for individual MS/MS spectra (*p* < 0.05) specified by MASCOT were accepted. Finally, all identifications were manually validated as previously described (Printz et al., 2013) and precursors not automatically selected were fragmented to increase the sequence coverage or to identify eventual co-migrating proteins. For this validation, the list of matched peptides was compared to the MS spectrum and in cases where high-intensity peaks were not matched to the identified protein secondary database, searches were performed. If these secondary searches resulted in the identification of a second protein, the spot was excluded from biological interpretation. Performing these secondary searches, and allowing the presence of semi-tryptic peptides during the database search, resulted in the identification of processed proteins. Further validation of close-to-threshold peptide identifications were done by looking at specific spectral features. Since the different oxidized forms of tryptophan are always found together, the identification of a peptide containing one of these oxidized forms was validated by looking at the presence of peaks corresponding to the same peptide with the other oxidation products of this residue (Trp; Trp +4 Da = kynurenin, Trp +16 Da = oxidized Trp; Trp +32 Da = N-formylkynurenin). Other easy-to-recognize spectral features that were looked at include the presence of a peak corresponding to the C-terminal arginine and the presence of the neutral loss of 64 Da from peptides containing oxidized methionine. Furthermore, spectral characteristics related to the presence of the certain residues (most notably proline and aspartic acid) were analyzed (Breci et al., 2003; Paizs and Suhai, 2005). Similar for the presence of the amino acid glutamine at the N-terminus of a peptide that is generally accompanied with the same peptide at −17 Da due to the formation of pyro-glutamic acid Functional annotation for proteins of unknown function was performed using the InterProScan tool (Jones et al., 2014; Finn et al., 2017). The mass spectrometry proteomics data have been deposited to the ProteomeXchange Consortium via the PRIDE (Vizcaíno et al., 2014) partner repository with the dataset identifier PXD006995.

### Immunoblotting

Two-dimensional (2-D) gel electrophoresis was performed using 7 cm strips (pH 4–7) as previously described, using indicated amounts of IV30, DMC or rat urine isolated leptospires. For immunoblotting, samples were transferred to Immobilon-P transfer membrane (Millipore, Bedford, MA) and blocked with 5% (w/v) non-fat dried milk in phosphate-buffered saline–0.1% (v/v) Tween 20 (PBS-T). Membranes were individually incubated with indicated antisera (anti-LipL32 or anti-LipL41) at 1:2,500 in PBS-T for 1 h at room temperature, or anti-trimethyllysine or anti-acetyllysine (PTM Biolabs, IL, U.S.A.) at 1:1,000 in 1% (w/v) non-fat dried milk in PBS-T overnight at 4°C, followed by incubation with horseradish-peroxidase anti-rabbit immunoglobulin G conjugate. Bound conjugates were detected using the SuperSignal WestPico substrate (Pierce) or Clarity Western ECL substrate (BioRad) and images acquired using a UVP Biospectrum–AC w/Bio Chemi camera (Cambridge, United Kingdom) or Bio-Rad ChemiDoc MP imaging system. The specificity of the PTM-specific antibodies has been previously reported (Chu et al., 2016; Hong et al., 2016).

## Results

### Pathogenic leptospires modify protein abundance in response to mammalian host signals

**Figure 1:**
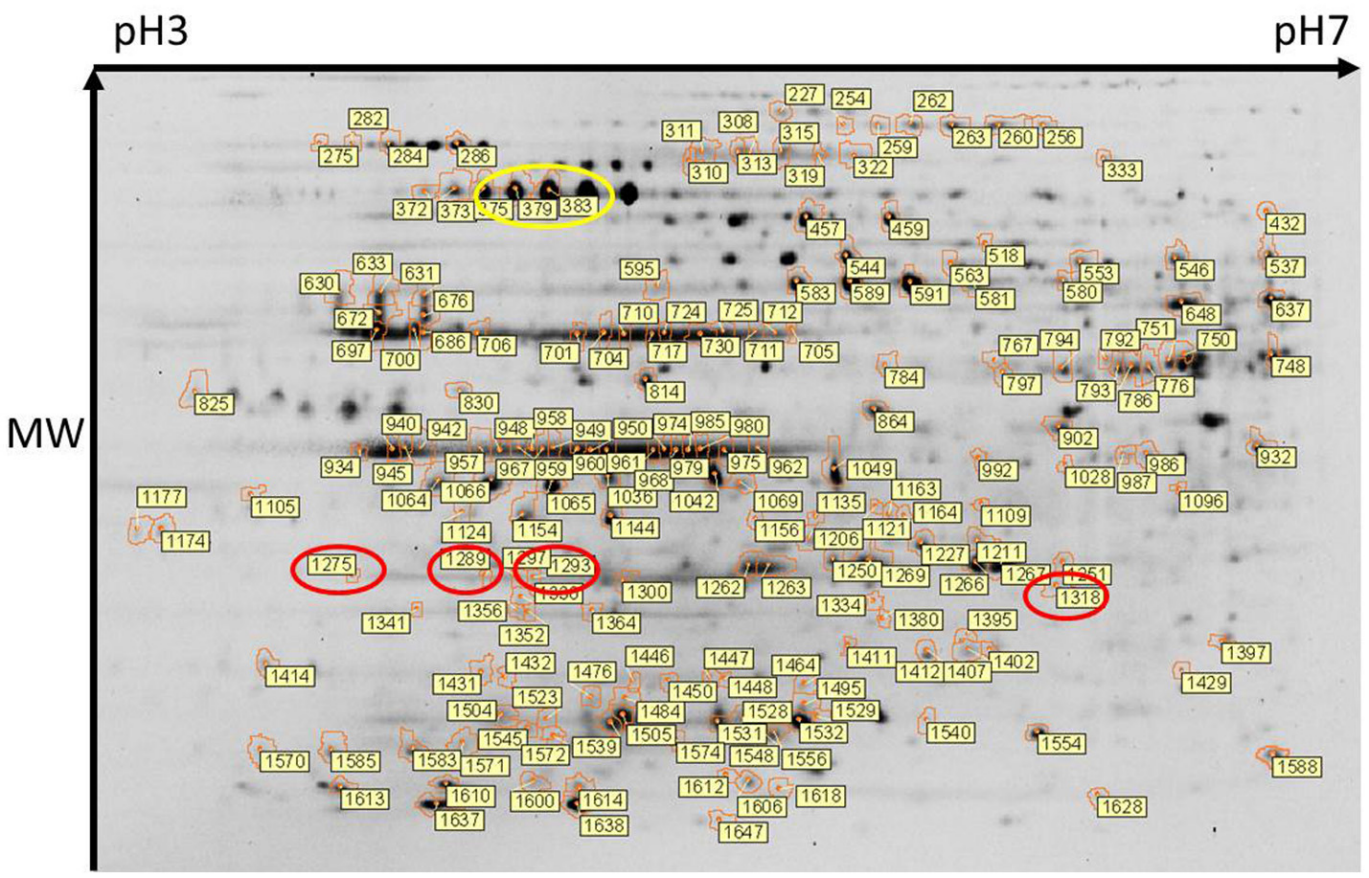
Two-dimensional gel electrophoresis of leptospires. Composite image of protein spots detected across all 9 gels and in which 1735 protein spots were aligned. Of these, 202 protein spots were determined to be differentially expressed (DE) (*p* < 0.05). DE protein spots, and their identifier (as listed in Supplementary Tables 1, 2) are indicated. Protein isoforms of the differentially expressed Loa22 and GroEL are circled in red and yellow respectively. An unmarked version of the stained gel is presented in Supplementary Figure 1.

**Figure 2:**
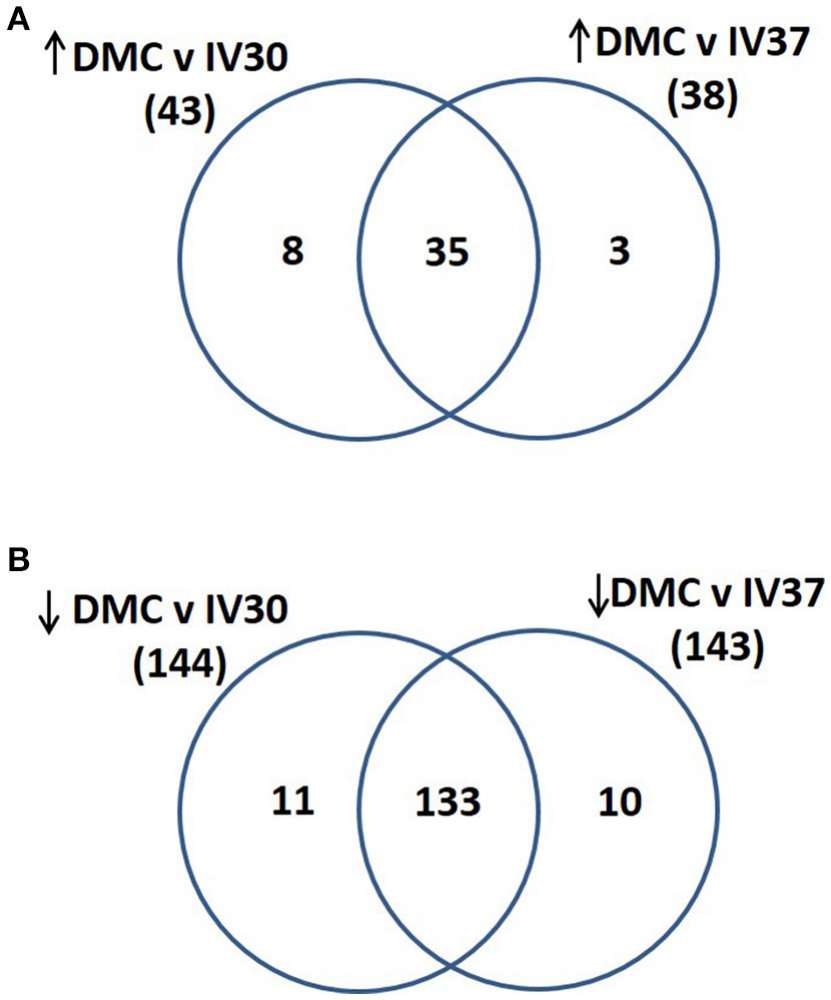
Venn diagram illustrating the numbers of proteins that were identified as **(A)** increased in abundance in DMC compared to IV30 or IV37 leptospires or **(B)** decreased in abundance in DMC compared to IV30 or IV37 leptospires.

Total protein profiles of leptospires cultivated *in vitro* at 30°C (IV30), 37°C (IV37) or in DMCs were compared by 2D-DIGE over a pH range of 3–7 NL, Figure 1. Of 1735 protein spots aligned across 9 gels comprising 27 scans, 202 protein spots were determined to be differentially expressed (*p* < 0.05, fold change >1.25 or < −1.25) across the three conditions (Supplementary Table 1). When DMC-cultivated leptospires were compared to IV30 leptospires, 187 spots were of different intensity (*p* < 0.05): of these, 43 were increased in DMCs whilst 144 were decreased (Supplementary Table 1). Similarly, when DMC-cultivated leptospires were compared to IV37-cultivated leptospires, 181 protein spots were differentially expressed (*p* < 0.05, fold change > 1.25 or < −1.25): 38 spots were increased in DMCs and 143 decreased. The majority of differentially expressed proteins are common to each group, Figure 2.

Comparison of IV30- and IV37-cultivated leptospires identified only 18 protein spots of significant different intensity (*p* < 0.05, fold change > 1.25 or < −1.25): seven were increased at 37°C and 11 were increased at 30°C.

### Protein identification

Spots of interest, as labeled in Figure 1, were excised from 2-D DIGE gel #4 for identification of proteins by mass spectrometry. Only those protein spots that had one single significant protein identification are discussed below. All protein identifications are provided in Supplementary Tables 1, 2. Proteins present at a higher abundance in DMCs compared to *in vitro*-cultivated leptospires included the known virulence factor Loa22 (OmpA-like domain membrane protein), previously shown to be of higher abundance in intact motile leptospires purified from the liver of experimentally-infected guinea pigs, or in leptospires excreted from the kidney of persistently infected rats (Nally et al., 2007b, 2011); four isoforms of Loa22 (protein spots 1,318, 1,275, 1,293, and 1,289, circled in Figure 1, Table 2, and Supplementary Tables 1, 2) were increased 1.9-, 1.75-, 1.58- and 1.44-fold in DMCs respectively, compared to IV30 leptospires. Similarly, the chaperone protein GroEL has also been shown to be increased in expression by leptospires excreted in urine from experimentally infected rats (Nally et al., 2011): three isoforms of GroEL (protein spots 375, 383, and 379, circled in Figure 1, Table 2, and Supplementary Tables 1, 2) were increased 3.95-, 2.59,- and 2.48-fold, respectively. These protein identifications serve as internal controls confirming the use of DMC-cultivated leptospires as a surrogate model for the characterization of protein expression by leptospires in response to mammalian host signals.

**Table 2:** Proteins more abundant (*p* < 0.05, fold > 1.25) in DMC-cultivated leptospires compared to IV30-cultivated leptospires.

**Protein spot number**	***p*-value**	**Av. ratio**	**GI accession number (Accession number)**	**Protein name (Locus tag)**	**InterPro scan analysis (for proteins of unknown function)**
313	0.00057	7.05	gi|45602612 (AAS72087.1)	methylmalonyl-CoA mutase (LIC_20058)	
992, 987	0.000076, 0.000011	4.49, 3.71	gi|45601096 (AAS70579.1)	succinate dehydrogenase iron-sulfur subunit (LIC_12003)	
375, 383, 379	0.0012, 0.017, 0.0015	3.95, 2.59, 2.48	gi|45600451 (AAS69936.1)	GroEL (LIC_11335)	
1,028	0.0046	3.53	gi|45602037 (AAS71516.1)	LipL41 (LIC_12966)	Tetratricopeptide repeat (TPR) domain
1,164	0.013	3.09	gi|45601580 (AAS71060.1)	3-hydroxybutyryl-CoA dehydratase (LIC_12495)	
282, 284	0.00071, 0.011	2.78, 2.07	gi|45599657 (AAS69145.1)	DnaK (LIC_10524)	
1,446, 1445	0.0052, 0.045	2.4, 1.75	gi|45600006 (AAS69493.1)	putative lipoprotein (LIC_10879)	None predicted
518	0.039	2.02	gi|45601495 (AAS70976.1)	putative glutamine synthetase protein (LIC_12407)	
1,318, 1,275, 1,293, 1,289	0.0037, 0.0098, 0.026, 0.024	1.9, 1.75, 1.58, 1.44	gi|45599329 (AAS68819.1)	Loa 22 (peptidoglycan associated cytoplasmic membrane protein) (LIC_10191)	OmpA-like domain

All the proteins that are of significant higher abundance in DMC-cultivated leptospires compared to *in vitro*-cultivated controls (IV30) are provided in Table 2 and Supplementary Tables 1, 2. Similar results are obtained when DMC leptospires are compared against IV30 or IV37 leptospires, Figure 2. The cobalamin-dependent methylmalonyl-CoA mutase was identified as the single protein (spot 313) that was most increased (7.05-fold change) in DMC-cultivated leptospires relative to IV30 leptospires (and increased 6.19-fold relative to IV37 leptospires). Additional metabolic enzymes increased in DMCs include succinate dehydrogenase iron-sulfur subunit (spots 992 and 987; increased 4.49- and 3.71-fold, respectively), 3-hydroxybutyryl-CoA dehydratase (spot 1,164; increased 3.09-fold) and glutamine synthetase (spot 518; increased 2.2-fold). Multiple isoforms of the chaperone protein DnaK were also increased in DMC-cultivated leptospires, as well as protein isoforms of a putative lipoprotein (LIC10879). A single protein isoform of the outer membrane lipoprotein LipL41 was identified as being increased in DMC-cultivated leptospires but this isoform (spot 1028) has an unusual mass and p*I* compared to that typically observed for LipL41 (see 7 isoforms identified below). This indicates that the protein species in spot 1,028 is a degradation product of the LipL41 protein, which corresponds in molecular mass and p*I* to an isoform of LipL41 that was previously identified in 2-D gels of leptospires cultivated at 37°C (Cullen et al., 2002).

Proteins found in higher amounts in samples from IV30- compared to DMC-cultivated leptospires are provided in Table 3 and Supplementary Tables 1, 2. Similar results are obtained when DMC leptospires are compared against IV30 or IV37 leptospires, Figure 2. Two isoforms of the molecular co-chaperone GroES (spots 1,638 and 1,637) were decreased in DMC-cultivated leptospires 12.47- and 9.45-fold, respectively. Two isoforms of the metabolic protein Elongation factor Tu (EF-Tu), which is hypothesized to be surface-exposed in leptospires and interact with both plasminogen and complement factor H (Wolff et al., 2013), were also decreased 10.7- and 2.81-fold, respectively. Three isoforms of peroxiredoxin (AhpC; spots 1,266, 1,263, & 1,262) were detected in lower amounts in DMC-cultivated leptospires, which is in contrast to the 5.96-fold increase in DMC for the corresponding gene transcript observed by RNAseq (Caimano et al., 2014). Consistent with lower levels of AphC protein, the levels of two isoforms of dihydrolipoamide succinyltransferase (SucB) were also decreased in abundance; this protein has been shown to interact with AphC to support antioxidant defense in *Mycobacterium tuberculosis* (Bryk et al., 2002). Proteins predicted to play a role in cell shape were found in lower amounts in DMC-cultivated leptospires and include the rod-shape determining protein MreB, cell shape determination protein LIC13483 and three isoforms of cell shape determination protein LIC12621. The expression of other proteins predicted to be involved in a wide range of cell functions was also diminished in DMC leptospires including proteins involved in RNA transcription and degradation (LIC11004, LIC13073, LIC12701, & LIC12636), amino acid metabolism (LIC20083, LIC13244, $& LIC10736), two component systems signal transduction systems (LIC12454 & LIC11194) and energy storage (LIC11241 & LIC11243).

**Table 3:** Proteins less abundant (*p* < 0.05, fold > −1.25) in DMC-cultivated leptospires compared to IV30-cultivated leptospires.

**Protein spot number**	***p*-value**	**Av. Ratio**	**GI Accession number (Accession number)**	**Protein name (Locus Tag)**	**InterPro scan analysis (for proteins of unknown function)**
1,638, 1,637	9.3E-07, 1.1E-07	−12.5, −9.45	gi|45600452 (AAS69937.1)	GroES (LIC_11336)	
591, 583	5.6E-07, 0.00022	−10.7, −2.81	gi|45601949 (AAS71428.1)	Elongation factor Tu (LIC_12875)	
1,266, 1,263, 1,262	1.9E-07, 0.00021, 0.0099	−9.25, −2.47, −1.8	gi|45600339 (AAS69825.1)	Peroxiredoxin (LIC_11219)	
631	1.2E-06	−8.01	gi|45600988 (AAS70471.1)	Putative lipoprotein (LipL46) (LIC_11885)	None predicted
1,042, 1,049	0.000026, 0.000072	−6.47, −5.23	gi|45599498 (AAS68987.1)	Electron transport flavoprotein beta subunit (LIC_10361)	
633, 630, 672, 676	0.00014, 0.00022, 0.00026, 0.00068	−6.08, −4.32, −3.47, −3.29	gi|45602121 (AAS71599.1)	Bacterial group 3 Ig-like protein (OmpL47) (LIC_13050)	
1,610, 1,613	0.000015, 0.00021	−6.08, −4.3	gi|45600128 (AAS69614.1)	Anti-sigma factor antagonist (LIC_11004)	
1,554	0.000011	−5.02	gi|45601701 (AAS71181.1)	Cell shape determination protein (LIC_12621)	
1,066, 1,545, 1,529	0.000023, 0.00096, 0.0059	−4.81, −2.60, −1.73	gi|45600754 (AAS70238.1)	Qlp42 = LipL45 (LIC_11643)	FecR protein
637	3.7E-06	−4.78	gi|45600094 (AAS69581.1)	Acyl-CoA dehydrogenase (LIC_10970)	
580, 581	6.5E-06, 2.9E-06	−4.69, −3.83	gi|45602637 (AAS72112.1)	S-adenosylhomocysteine hydrolase (LIC_20083)	
1,606	0.00012	−4.61	gi|45601539 (AAS71020.1)	Response regulator (LIC_12454)	
1,144	0.000059	−4.5	gi|45600133 (AAS69619.1)	Conserved hypothetical protein (LIC_11009)	None predicted
260, 256	0.0001, 0.0031	−4.14, −3.2	gi|45601779 (AAS71259.1)	Polyribonucleotide nucleotidyltransferase (LIC_12701)	
333	0.0015	−3.53	gi|45602455 (AAS71932.1)	Polysaccharide deacetylase (LIC_13392)	
780	0.00017	−3.42	gi|45600315 (AAS69801.1)	Putative citrate lyase (LIC_11194)	
1,174, 1,177	0.000066, 0.0047	−3.13, −1.72	gi|45600937 (AAS70420.1)	Putative lipoprotein (LIC_11834)	Fe (2+)-dicitrate sensor, transmembrane component, FecR protein
642	0.00052	−3.11	gi|45602311 (AAS71788.1)	Isocitrate dehydrogenase (LIC_13244)	
1,495	0.0071	−3.07	gi|45599867 (AAS69355.1)	Conserved hypothetical protein (LIC_10736)	Endoribonuclease L-PSP/chorismate mutase-like
1,548, 1,556	0.000052, 0.0029	−2.93, −2.07	gi|45601701 (AAS71181.1)	Cell shape determination protein (LIC_12621)	
1,250	0.000081	−2.92	gi|45600531 (AAS70016.1)	ATP-dependent Clp protease (LIC_11417)	
432	0.000084	−2.67	gi|45601716 (AAS71196.1)	Rho (LIC_12636)	
259	0.00035	−2.63	gi|45601070 (AAS70553.1)	Cyclic nucleotide binding protein (LIC_11977)	
962, 957, 974, 943, 960, 968, 969, 950, 979, 948, 949, 947, 975, 967, 940, 945, 959, 934, 958, 942	0.00054, 0.0016, 0.0012, 0.00081, 0.00091, 0.0017, 0.0094, 0.0027, 0.0024, 0.0019, 0.0023, 0.0017, 0.041, 0.0044, 0.0023, 0.027, 0.0038, 0.0013, 0.0054, 0.012	−2.52, −2.51, −2.48, −2.45, −2.33, −2.29, −2.23, −2.21, −2.20, −2.19, −2.17, −2.12, −2.11, −2.1, −2.09, −2.09, −2.07, −2.06, −1.98, −1.92	gi|45600468 (AAS69953.1)	LipL32 (LIC_11352)	
537, 546	0.00014, 0.00041	−2.50, −2.14	gi|45601560 (AAS71041.1)	Dihydrolipoamide succinyltransferase (LIC_12476)	
459, 227, 457	0.012, 0.03, 0.046	−2.40, −1.69, −1.56	gi|30652620 (Q72SY1.2)	ATP synthase subunit alpha (LIC_11241)	
1,612	0.00042	−2.38	gi|45602544 (AAS72021.1)	Cell shape determination protein (LIC_13483)	
814	0.0041	−2.17	gi|45600379 (AAS69864.1)	MreB (LIC_11258)	
254	0.0022	−2.13	gi|45601070 (AAS70553.1)	Cyclic nucleotide binding protein (LIC_11977)	
725, 717, 701, 711, 710, 706, 704	0.0068, 0.0035, 0.0067, 0.034, 0.011, 0.0039, 0.05	−2.07, −1.87, −1.79, −1.67, −1.61, −1.60, −1.45	gi|45602037 (AAS71516.1)	LipL41 (LIC_12966)	TPR domain
544	0.014	−2.06	gi|45600364 (AAS69849.1)	ATP synthase beta chain (LIC_11243)	
286	0.037	−2.03	gi|45599657 (AAS69145.1)	DnaK (LIC_10524)	
1,364, 1,352	0.02, 0.0081	−1.78, −1.55	gi|304570487 (AAS69491.1)	Cytochrome C/hypothetical protein (LIC10877)	
1,251	0.028	−1.71	gi|45602144 (AAS71622.1)	Transcriptional regulator (TetR family) (LIC_13073)	

The abundance of several known (or putative) outer membrane (OM) proteins and lipoproteins and conserved hypothetical proteins were also down in DMC-cultivated leptospires including LipL46 (LIC11885), OmpL47 (LIC13050), Qlp42/LipL45 (LIC11643), LipL32 (LIC11352), and LipL41 (LIC12966). Of note, 20 isoforms of LipL32 were all down in DMC. Similarly, the intensity of 7 spots wherein LipL41 is identified was down. Collectively, the identification of multiple isoforms of LipL32 and LipL41 derived from DMC leptospires which were all shifted in the same direction on a 2-D gel prompted us to explore the possibility that this shift was mediated via PTM. However, analysis of MS-spectra of different spots wherein the same protein was identified failed to identify MS spectral features that could be identified as modified peptides, likely a reflection of the limited amounts of protein used in 2-D DIGE gels.

### Protein post-translational modifications

**Figure 3:**
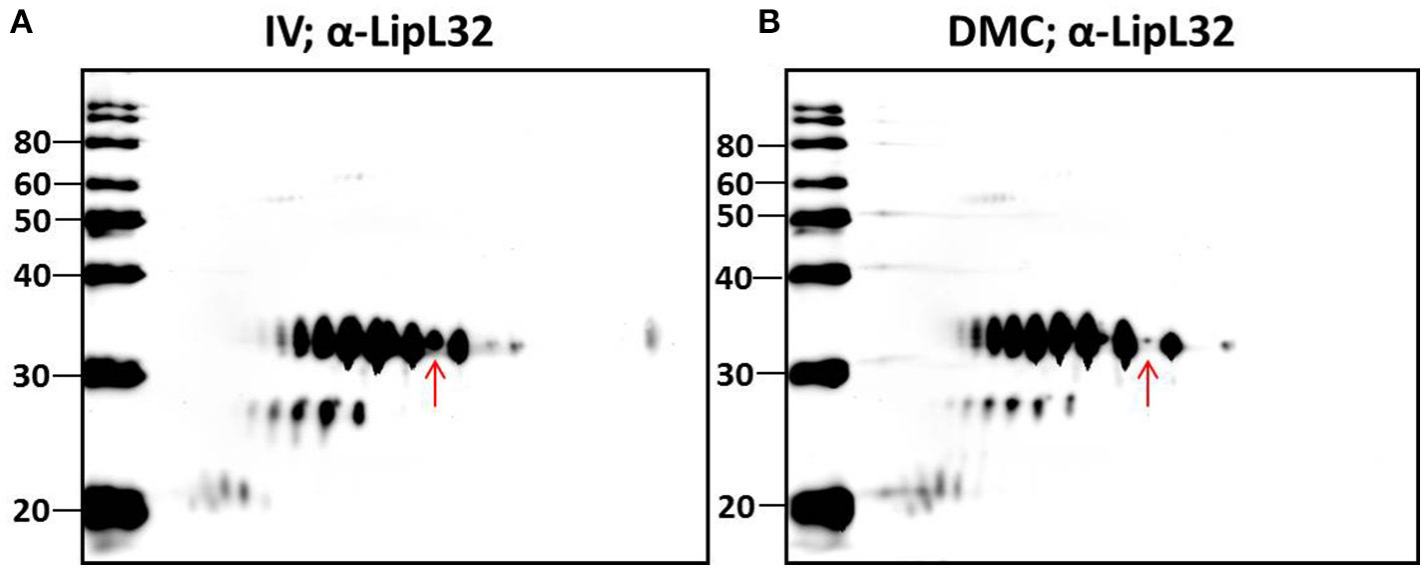
2-D Immunoblot analysis of **(A)** IV- and **(B)** DMC-cultivated leptospires. Approximately 10^7^ IV or DMC cultivated leptospires (strain Fiocruz L1-130) were separated by 2-D gel electrophoresis and probed with antiserum specific for LipL32. Red arrow indicates the same protein isoform in each immunoblot of LipL32 when immunoblots are overlaid. Molecular mass markers (kDa) are indicated.

Multiple protein species of a number of individual proteins were identified. This was exemplified by LipL32, the most abundant leptospiral outer membrane protein; 20 LipL32-isoforms were of lower abundance (< −1.25-fold, *p* < 0.05) in DMC leptospires (Table 3 and Supplementary Tables 1, 2). 2D-immunoblotting of DMC- and IV30-cultivated leptospires with LipL32-specific antiserum confirms reactivity with multiple protein isoforms, but results of immunoblot suggest that DMC- and IV30-cultivated leptospires express roughly equivalent amounts of LipL32 (Figures 3A,B). However, a shift in the ratio between the isoforms at different isoelectric points is observed, as indicated by an arrow in Figures 3A,B.

Characterization of the intact mass of LipL32 derived from purified OM vesicles confirmed that proteoforms of LipL32 can differ in their fatty acid moieties that anchor this lipoprotein into the outer membrane (i.e., lipoforms; Nally et al., 2005). More recently, studies have demonstrated that PTM occur on lysine residues of LipL32 during renal excretion compared to *in vitro*-cultivated controls (Witchell et al., 2014). Thus, we hypothesized that specific PTMs could account for, at least in part, the observed shift in p*I* of isoforms of LipL32 in DMC- compared to IV30-cultivated leptospires as detected by 2-D DIGE, and that such modifications are in response to environmental cues encountered during host infection.

**Figure 4:**
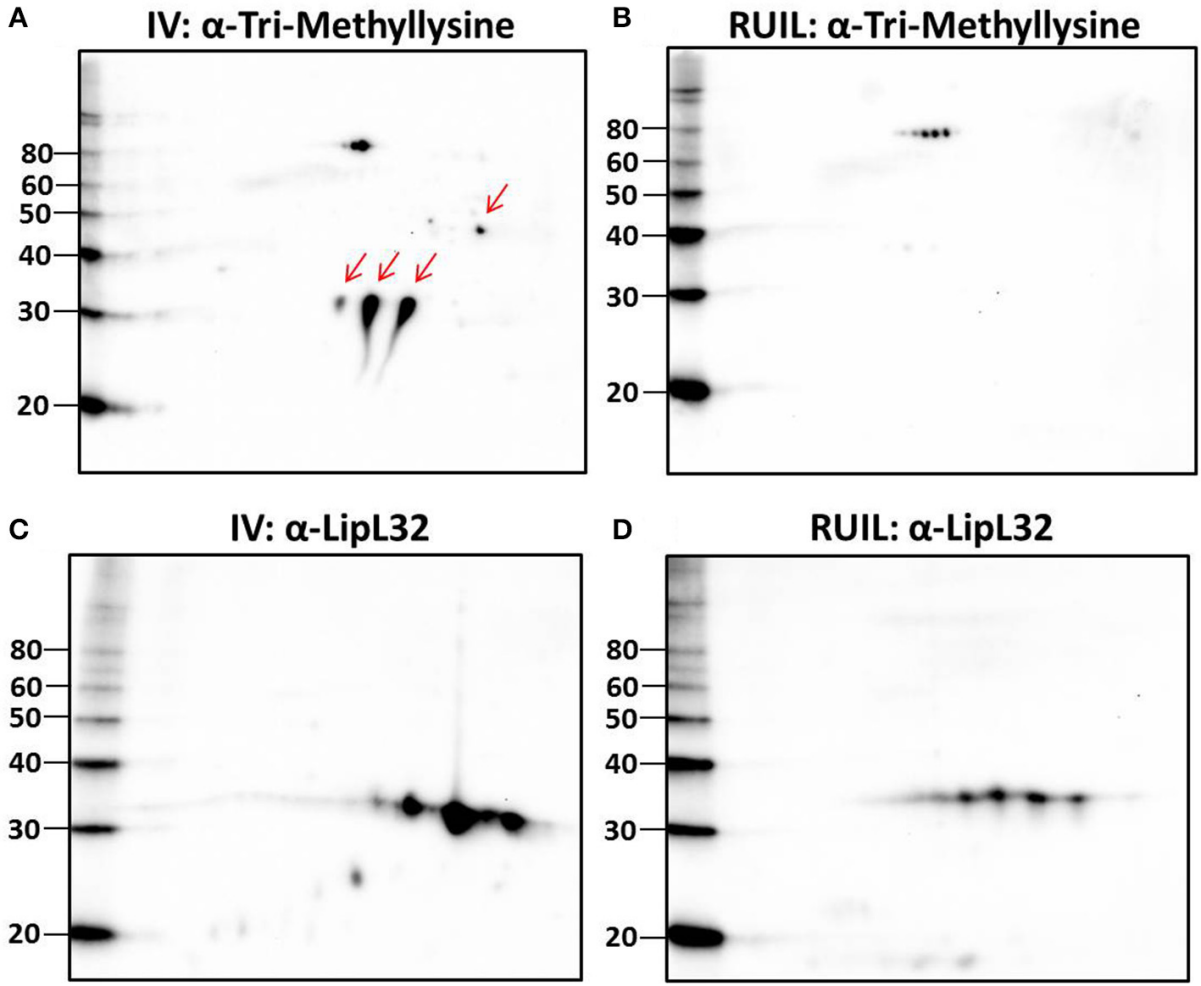
2-D Immunoblot analysis of IV and rat urine isolated leptospires. Approximately 2 × 10^7^ IV30 **(A,C)** or rat urine isolated leptospires (RUIL) (Strain RJ19115) **(B,D)** were separated by 2-D gel electrophoresis (pH 4–7) and probed with antiserum specific for trimethyllysine **(A,B)** or antiserum specific for LipL32 **(C,D)**. Arrows indicate antigens reactive with anti-trimethyllysine that are expressed by IV- but not DMC-cultivated leptospires. Molecular mass markers (kDa) are indicated.

**Figure 5:**
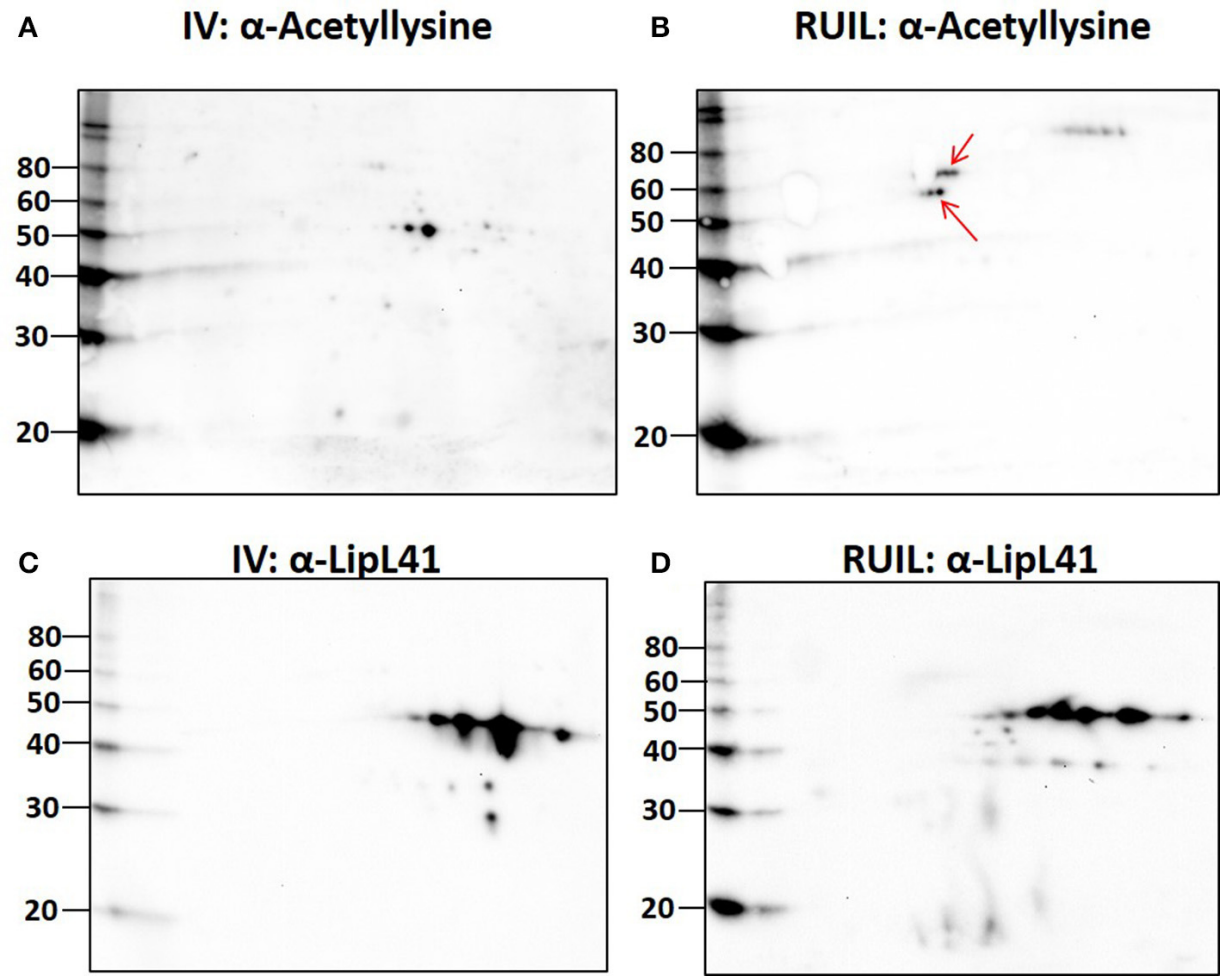
2-D Immunoblot analysis of *in vitro* (30°C; IV) and rat urine isolated (RUIL) leptospires. Approximately 2 × 10^7^ or Strain RJ19115 leptospires were separated by 2-D gel electrophoresis and probed with antiserum specific for acetyllysine **(A,B)** or LipL41 **(C,D)**. Arrows indicate antigens reactive with anti-acetyllysine that are expressed by DMC- but not IV30-cultivated leptospires. Molecular mass markers (kDa) are indicated.

To test this hypothesis, urinary derived leptospires were collected from experimentally infected rats for 2-D immunoblotting with antisera specific for the detection of defined protein PTM. Experimental infection of rats was performed with an alternative strain (strain RJ19115) to ensure that significant numbers of urinary derived leptospires could be collected for analysis by 2-D immunoblot. Immunoblotting confirmed that several protein antigens from IV30 were reactive with anti-trimethyllysine (Figure 4A, arrows), which were not readily detected in rat urine isolated leptospires (Figure 4B). Reactive antigens include several isoforms with similar mass and p*I* to that of LipL32 (Figures 4C,D). Additionally, immunoblotting with antibody specific for the protein PTM acetyllysine provides further evidence for differential expression of PTM by leptospires excreted in urine compared to their *in vitro*-cultivated counterpart (Figures 5A,B).

## Discussion

The transcriptome and proteome of leptospires cultivated *in vitro* has been studied extensively. However, comparative analysis of the transcription and translation of leptospiral outer membrane proteins, in response to increased temperature, identified a large number of differences in protein profile without a corresponding change in transcript levels (Lo et al., 2009). These results highlight the need to characterize the contribution of post-transcriptional regulatory pathways to mammalian host adaption by pathogenic leptospires. We have used the DMC model to comprehensively identify gene expression of leptospires *in vivo* by RNA-seq (Caimano et al., 2014). In the current study, we applied the DMC model to identify proteins that are differentially abundant in response to mammalian host signals. Our analysis confirms that pathogenic leptospires modulate protein expression in response to growth in DMC. However, and similar to previous studies comparing differential expression of gene transcription and translation in leptospires (Lo et al., 2009), none of the identified differentially abundant proteins had equivalent differentially expressed gene transcripts (based on our published RNA-seq data; Caimano et al., 2014).

Pathogenic leptospires are highly fastidious bacteria which are typically cultured in albumin enriched media at 28–30°C (Zuerner, 2005). This is in stark contrast to conditions encountered during disease transmission; leptospires adapt to and replicate in the renal tubule of a reservoir host from which they are excreted via urine to survive in suitable moist environments. After host invasion, they disseminate haematogenously to multiple organs (Coutinho et al., 2014), and in particular, the kidney, to establish and maintain persistent infection (Bolin and Alt, 2001; Athanazio et al., 2008). To further understand pathogenic mechanisms of leptospirosis and identify gene expression pathways and regulatory mechanisms predicted to facilitate host infection, prior studies have relied on modification of *in vitro* growth conditions to emulate conditions encountered during disease transmission and within the mammal (Matsunaga et al., 2013). Since pathogenic leptospires are not readily amenable to targeted genetic manipulation, validation of gene encoded virulence factors is generally dependent on random mutagenesis (Murray et al., 2009). Such is the case for *loa22*, the first virulence factor of leptospires identified that satisfied molecular Koch's postulates (Ristow et al., 2007). However, transcript levels of *loa22* do not change in response to temperature (Lo et al., 2006; Qin et al., 2006), osmolarity (Matsunaga et al., 2007), serum concentration (Patarakul et al., 2010) or growth in DMC (Caimano et al., 2014), but are down-regulated during interaction with macrophages (Xue et al., 2010). In contrast, the protein expression of Loa22 is significantly increased in leptospires extracted from the liver tissue of acutely infected guinea pigs (Nally et al., 2007b), and increased 2.1-fold in leptospires excreted from persistently infected rats (Monahan et al., 2008; Nally et al., 2011). In addition, levels of Loa22 are increased in leptospires cultivated in iron-depleted media and the presence of serum (Eshghi et al., 2009). Here, we report that multiple proteoforms of the Loa22 virulence factor and outer membrane protein are increased 1.9-, 1.75-, 1.58-, and 1.44-fold (spot 1,318, 1,275, 1,293, & 1,289, respectively) in response to mammalian host signals encountered by leptospires cultivated in DMC, a model system for the cultivation of leptospires *in vivo*, Figure 1.

Previously we have shown that the gene expression for peroxiredoxin (*ahpC*) was increased 5.96-fold in DMC-cultivated leptospires compared to IV30 leptospires. This contrasts sharply with our current findings in which three protein isoforms of AhpC were decreased 9.25-, 2.47-, and 1.8-fold in DMC compared to IV30 leptospires, and in two isoforms decreased 6.26- and 2.01-fold in DMC compared to IV37 leptospires. Recent studies comparing leptospires cultivated at 37°C compared to 30°C demonstrated that AhpC is increased 1.6-fold without a corresponding increase in gene transcript (Lo et al., 2009). Collectively, these results suggest that AhpC is subject to, and as yet uncharacterized, post-transcriptional regulation. *L. interrogans* does not encode an AhpF, the usual reducing partner for AhpC. In *M. tuberculosis*, AhpC interacts with SucB (dihydrolipoamide succinyltransferase), which was detected at lower levels in DMC leptospires compared to IV30 leptospires (−2.5 and −2.14-fold) or compared to IV37 leptospires (−2.49 and −2.01-fold).

The cobalamin (vitamin B_12_) dependent methylmalonyl-CoA mutase was identified in spot 313 with a fold change of 7.05 and 6.19 in DMC-cultivated leptospires compared to IV30 or IV37 leptospires, respectively. This metabolic enzyme was also identified in spots 319 and 315 which were increased 10.48- and 6.8-fold respectively in DMC leptospires compared to IV30. Recently identified small non-coding RNAs include cobalamin riboswitches which are expressed by DMC- cultivated leptospires (Ricaldi et al., 2012a; Caimano et al., 2014; Zhukova et al., 2017). These function as cis-regulatory elements in 5′-untranslated regions of vitamin B_12_-related genes, however their function remains to be determined. Comparative analysis of 20 species of *Leptospira* predicted that only pathogenic strains make cobalamin *de novo* from L-glutamate, suggesting that this process is critical *in vivo*, and that such autotrophy allows leptospires to infect mammals in the face of vitamin B_12_ sequestration by the host (Fouts et al., 2016).

As a general trend, and similar to protein expression by leptospires cultivated at 37°C compared to 30°C (Lo et al., 2009), the abundance of many OM proteins was reduced in DMC compared to IV30 and IV37 leptospires. LipL46, a surface-exposed lipoprotein expressed during leptospiral dissemination in the mammalian host (Matsunaga et al., 2006), was downregulated 8.01- and 4.37-fold in DMC compared to IV30 and IV37 leptospires. No change in transcript levels for *lipL46* was observed in DMC or leptospires cultured at 37°C (Lo et al., 2006; Qin et al., 2006; Caimano et al., 2014), though expression of this gene has been shown to be downregulated when interacting with macrophages (Xue et al., 2010).

Four isoforms of OmpL47 (LIC13050) were 6.08-, 4.32-, 3.47-, and 3.29-fold lower in DMC leptospires compared to IV30 leptospires; similarly the same four isoforms were down 3.24-, 2.43-, 2.28-, and 2.3-fold when compared to IV37 leptospires. However, no difference was detected at the level of gene transcription by RNAseq (Caimano et al., 2014). The observed fold change of OmpL47 is similar to previous studies that demonstrated that OmpL47 is a temperature-regulated protein and of lower abundance in leptospires cultured at 37°C compared to those cultured at 30°C, and with no corresponding change in gene expression (Lo et al., 2009). In agreement with this, our analysis indicates that temperature is an important environmental cue for the expression of OmpL47 since four isoforms (spots 633, 630, 672, and 676) were of lower abundance (1.88-, 1.78-, 1.52-, and 1.43-fold, respectively) when IV37- were compared only with IV30-cultivated leptospires (Supplementary Table 1). Nevertheless, OmpL47 is reported to be a surface exposed protein that binds skin and elastin and is expressed during infection (Eshghi et al., 2009; Pinne et al., 2010). OmpL47 has also been detected in cell culture supernatants so it's apparent down-regulation may be due to increased levels of secretion (Zeng et al., 2013; Eshghi et al., 2015).

LipL45 was originally identified as Qlp42, the abundance of which was increased in leptospires cultivated at 37°C compared to 30°C without an apparent change in the levels of gene transcription (Nally et al., 2001a; Lo et al., 2009). In contrast to a predicted molecular mass of 39.8 kDa for LipL45/Qlp42, the actual mass was measured as 24,811 and 26,461 Da consistent with a 30 kDa doublet observed on sodium dodecyl sulfate-polyacrylamide gel electrophoresis gels and processing of the N-terminus of the mature protein (Nally et al., 2001a, 2005; Matsunaga et al., 2002). We identified multiple isoforms of LipL45 that were detected at lower levels in DMC compared to IV30 or IV37 leptospires. InterproScan identified LipL45 as a FecR protein which is involved in the regulation of iron dicitrate transport. In the absence of citrate, FecR inactivates the probable RNA polymerase sigma factor, FecI. FecR is likely a sensor for periplasmic iron dicitrate. Gene expression levels of *lipL45* are down over 5-fold during interaction with macrophages (Xue et al., 2010). LIC11834, which encodes Lsa33, was also predicted to be a FecR protein and was similarly down in DMC leptospires. This protein binds laminin and activates plasminogen, and is predicted to be surface exposed (Domingos et al., 2012). Gene expression of *lsa33* is down-regulated −2.03-fold at physiological osmolarity compared with low osmolarity (Matsunaga et al., 2007).

Unexpectedly, 20 protein isoforms of LipL32 were uniformly identified by 2-D DIGE as differentially expressed. Similarly, 7 isoforms of LipL41 were differentially expressed. Both LipL32 and LipL41 are constitutively expressed under a wide range of *in vitro* and *in vivo* conditions, though their functions have yet to be determined (Shang et al., 1996; Haake et al., 2000). LipL32 and LipL41 are outer membrane proteins and multiple isoforms are readily observed in 2-D gels of whole or OM enriched leptospires, either by direct protein staining or immunoblotting (Nally et al., 2005, 2007b), as illustrated by the detection of LipL32 in DMC or IV30 leptospires (Figures 3A,B). Collectively, the apparent shift in p*I* of each of these isoforms could be explained, in part, by PTM. Multiple PTM have been detected on proteins of saprophytic and pathogenic leptospires, and on specific proteins, including OmpL32 and LipL32 which are reported to be regulated, at least in part, by exposure to mammalian host conditions encountered during infection (Eshghi et al., 2012; Witchell et al., 2014). To test this hypothesis further, leptospires were collected directly from the urine of experimentally infected rats and compared to IV30 leptospires by 2-D immunoblotting with antiserum specific for the PTM trimethyllysine and acetyllysine (Figures 4, 5). The quantity of leptospires shed in the urine of experimentally infected rats is dependent on multiple factors including route of inoculation, dose, time post-infection, and strain (Athanazio et al., 2008; Bonilla-Santiago and Nally, 2011; unpublished observations). The use of an alternative strain for experimental infection of rats served to not only ensure that significant numbers of urinary derived leptospires could be collected for analysis by 2-D immunoblot, but also to ensure that our observations were relevant to more than one strain, and during actual infection as predicted by our model system for the cultivation of leptospires *in vivo* (Caimano et al., 2014). Collectively our results highlight that differences in protein modification with trimethyllysine and acetyllysine were observed between urinary vs. IV derived leptospires, indicating that these modifications were regulated, at least in part, by mammalian host signals. 2-D immunoblotting also suggested that LipL32 from IV30 leptospires contained trimethyllysine whilst urinary-derived leptospires did not; a finding which differs from the recent identification of trimethyllysine by mass spectrometry in urinary derived LipL32. The reasons for this are not yet clear and may be due to differences in modification potential of different strains of leptospires used in DMCs (Strain Fiocruz L1-130) compared to experimental infection (Strain RJ19115). In any case, a comprehensive analysis of protein structures of LipL32 as expressed during host infection is warranted. It has been hypothesized that protein PTM by leptospires may be dependent, at least partially, on elevated bacterial density (Witchell et al., 2014). Our analysis of DMC-cultivated leptospires is limited to those explanted at ~10 days; this time-point ensures recovery of sufficient motile leptospires (up to 3 × 10^8^ per ml) required for proteomics; this cell density in DMC corresponds to late logarithmic phase. Additional studies will be required to determine if cell density, similar to that encountered during persistent renal colonization, influences PTM. Similarly, given that DMC-cultivated leptospires are shielded from the host's immune system, it will be important to determine whether some PTMs occur in response to environmental (or physiological) stimuli vs. immune pressures encountered during persistent renal colonization.

In contrast to transcriptomic analyses which have the potential to determine the gene expression for all genes within a bacterium, gel based proteomics has inherent limitations. The genome of *L. interrogans* encodes more than 3,500 proteins (Nascimento et al., 2004), 69% of which have a theoretical p*I* > 7.0 (Nally et al., 2007a). Our analyses were limited to those proteins with a p*I* of 3–7 since previous research has demonstrated that antigens reactive with convalescent sera are expressed within this range (Kositanont et al., 2007). Under these conditions, more than 1,700 protein spots were detected across all biological replicates of DMC- or IV-cultivated leptospires. The sensitivity of gel-based proteomics is often limited to the most abundantly expressed proteins and this is reflected in those proteins we identified, given that whole leptospires were used. For example, the genes encoding DnaK, LipL32, GroEL, Elongation Factor Tu, and LipL41, are amongst the most transcribed genes in either DMC or IV leptospires (Caimano et al., 2014), and these were readily identified at the protein level. In contrast, those genes which are most differentially expressed between DMC and IV30 leptospires have (generally) an order of magnitude fewer transcripts (Caimano et al., 2014). In future work, we aim to increase sensitivity for the detection of outer membrane proteins, and their respective PTM, that interact with the host during infection; e.g., by enrichment of OM proteins with TX-114, and as required to detect the differential expression of the OM lipoprotein LipL36 in response to temperature (Nally et al., 2001b). Advantages of gel based proteomics include the ability to identify PTM associated with specific protein isoforms, and to perform immunoblots to determine which spots are reactive with serum from exposed animals (or other ligands of interest).

Post-translational methylation and acetylation have been implicated in protein activity, resistance to proteolysis, virulence (Calder et al., 2015), phase variation of pilin, type III secretion, chemotaxis, and motility (Kort et al., 1975; Barak and Eisenbach, 2001), stress responses (Ma and Wood, 2011), and metabolism (Ouidir et al., 2015). Bacterial pathogens express OM proteins which can contain multiple PTM, and interact directly with the host during infection. Whilst generally accepted that PTM modify protein function, studies investigating the consequences of regulating these in response to host infection are limited (Cain et al., 2014; Grangeasse et al., 2015). In pathogenic leptospires, PTM of outer membrane proteins are predicted to facilitate immune evasion, and thus persistence (Witchell et al., 2014). Our results advocate that differential protein PTM, including methylation and acetylation, are regulated in response to infection. Collectively, our results highlight the need to further examine regulatory processes employed by pathogenic leptospires to adapt to the host during infection. Our analyses confirm that leptospires cultivated within DMCs, in response to mammalian host conditions, are amenable to genomic, transcriptomic and proteomic analysis and that DMC leptospires can be used to define such regulatory pathways. Finally, our results highlight the need to consider how leptospires modify their proteins with PTM, as this can influence the success of candidate vaccine and diagnostic antigens.

## Author contributions

Conceived and designed the experiments: JN, MC. Performed the experiments: JN, AG, SP, KS, JR, MC. Contributed resources/reagents/materials/analysis tools: JN, JR, JS, AM, MC. Wrote the paper: JN. Revised the paper: JN, AG, SP, KS, JR, JS, AM, MC.

### Conflict of interest statement

The authors declare that the research was conducted in the absence of any commercial or financial relationships that could be construed as a potential conflict of interest.

## Supplementary material

Supplementary Figure 1 Two-dimensional gel electrophoresis of leptospires. As shown in Figure 1 but without labels.

Supplementary Table 1 List of all differentially expressed proteins.

Supplementary Table 2 Mass spectrometry data for all protein identifications. Search parameters are provided in the parameters sheet. The summary sheet provides all significant hits which are highlighted in green, and proteins spots with more than one significant identification are highlighted in yellow. Proteins identifications that were returned as hypothetical or unknown were subject to BLAST (Altschul et al., 1990) and these identifications are italicized.
