# Supplementary figures and images for: Pathogenic Leptospires Modulate Protein Expression and Post-translational Modifications in Response to Mammalian Host Signals
Source: Front Cell Infect Microbiol. 2017 Aug 9;7:362. doi: 10.3389/fcimb.2017.00362 (PMC5553009; doi:10.3389/fcimb.2017.00362)

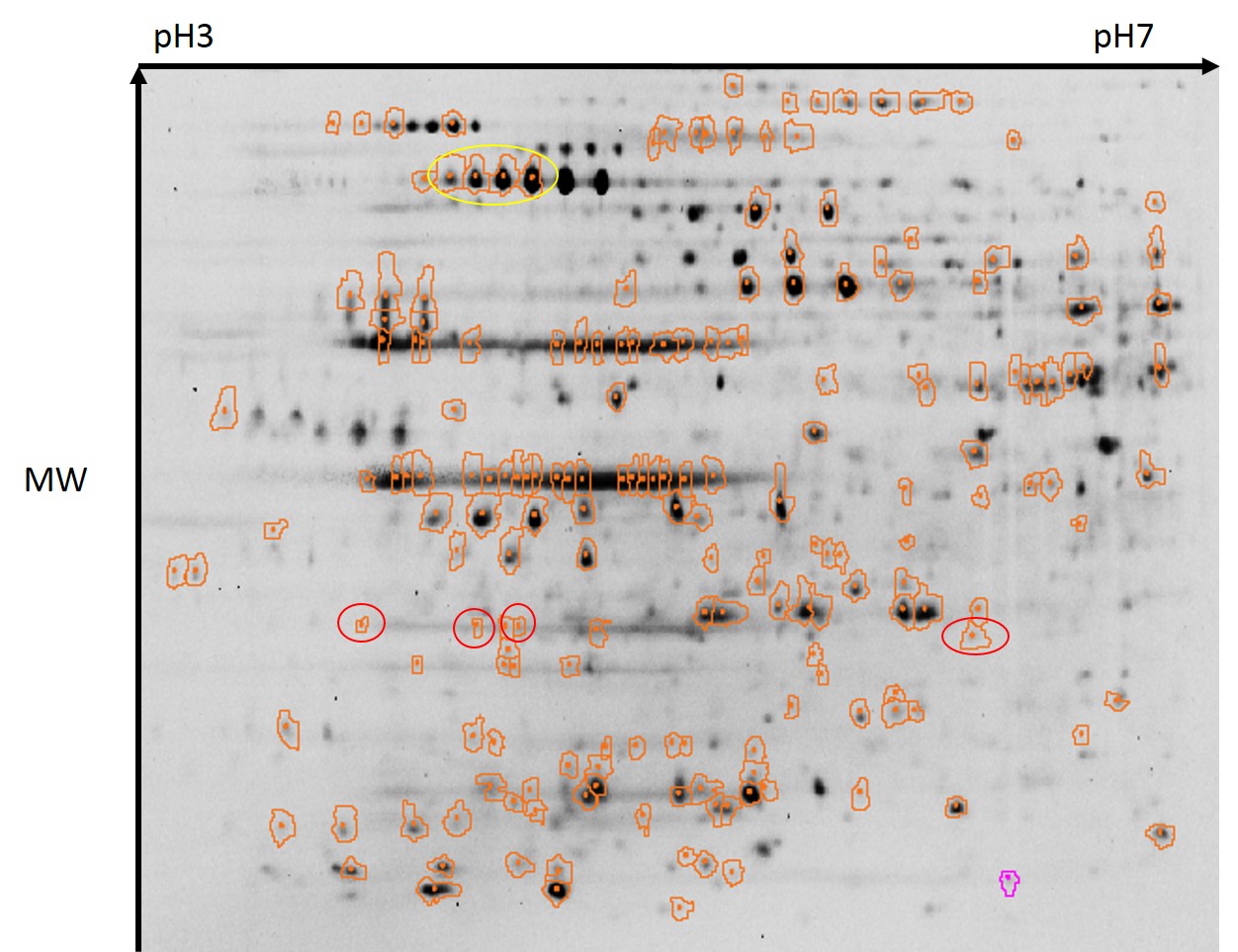

Supplement: Supplementary Figure 1 — Two-dimensional gel electrophoresis of leptospires. As shown in Figure 1 but without labels. [file Image1.JPEG]
